# Efficacy of Radiotherapy in Patients with Relapsing Primary Rosai–Dorfman Disease of the Nasal Cavity

**DOI:** 10.3390/medicina61040585

**Published:** 2025-03-25

**Authors:** Caius-Codrut Sarafoleanu, Florentina-Carmen Badea, Alina-Maria Georgescu, Gabriela-Cornelia Musat, Anica Andrei, Ionut Tanase

**Affiliations:** 1 Department of Otorhinolaryngology, “Carol Davila” University of Medicine and Pharmacy, 050474 Bucharest, Romania; codrut.sarafoleanu@umfcd.ro (C.-C.S.); gabriela.musat@umfcd.ro (G.-C.M.); 2 Department of Otorhinolaryngology, “Sfanta Maria” Clinical Hospital, 011172 Bucharest, Romania; 3 Department of Otorhinolaryngology, Medicover Hospital, 020331 Bucharest, Romania; carmeen.badea@gmail.com; 4 Doctoral School, University of Medicine and Pharmacy of Craiova, 200349 Craiova, Romania; 5 Department of Laboratory Medicine, Victor Babes Clinical Hospital of Infectious Diseases and Pneumophthisiology, 200515 Craiova, Romania; 6 Department of Radiotherapy, Radiation Therapy Center Amethyst, 075100 Otopeni, Romania; dr.andrei.anica@gmail.com

**Keywords:** Rosai–Dorfman disease, histiocytosis, radiotherapy, sinonasal, S100 protein

## Abstract

Rosai–Dorfman disease (RDD) is a rare proliferative disorder characterized by an overproduction of a type of immune cell called histiocytes, with unknown etiology. Although extranodal involvement is not uncommon, it is rarely seen within the nose. The available data are limited, and currently, there are no established guidelines for managing RDD. Different therapeutic options have been described, including corticosteroids, surgery, radiotherapy, or chemotherapy. This study aims to evaluate the potential benefit of radiotherapy in patients with nasal Rosai–Dorfman disease to improve the current diagnostic and therapeutic management. Herein, we report the case of a 54-year-old female patient with nasal RDD refractory to systemic corticosteroid therapy and transnasal endoscopic resection. She received fractionated low-dose radiotherapy with a total dose of 30 cGy administered in 2 cGy daily fractions. Complete remission was achieved, highlighting the potential role of radiotherapy as an effective therapeutic option for relapsing or steroid-refractory cases. This is one of the few well-documented cases reported with nasal Rosai–Dorfman disease treated with radiotherapy. Ongoing research on novel therapies offers hope for improved outcomes in relapsing cases that fail to respond to conventional treatments.

## 1. Introduction

Rosai–Dorfman disease (RDD) is a rare and benign histiocytic disorder that primarily affects lymph nodes, most commonly in the neck, but can also manifest extranodal involvement. It was first described in 1965 by Destombes [1], and then in 1969 by two pathologists from Yale University and Stanford University, Rosai and Dorfman, respectively, who described this disease in a series of four patients as sinus histiocytosis with massive lymphadenopathy (SHML) [2].

Based on the extent of involvement, there are three entities: nodal, extranodal, and cutaneous RDD. Since 2016, according to the revised histiocyte classification by the Histiocyte Society, the non-cutaneous forms of RDD have been placed into the “R” group of histiocytosis, whereas the cutaneous form is considered a separate entity within the “C” group [3].

Patients with RDD typically present with massive, painless cervical lymphadenopathy. This enlargement of the lymph node is usual bilateral and may be accompanied by systemic symptoms such as fever, weight loss, and night sweats. While the disease predominantly involves the lymph nodes, particularly in the cervical region, extranodal manifestations are reported in approximately 43% of cases. The skin, eyes, and bone structures are the most commonly affected extranodal sites. Involvement of the nasal cavity and paranasal sinuses is rare (11%) [4]. Nasal RDD can mimic other common conditions such as chronic rhinosinusitis, nasal polyps, or even neoplastic processes. Patients often present with nonspecific symptoms such as nasal obstruction, epistaxis, nasal deformity, or facial swelling, which can lead to delayed or incorrect diagnoses. The diagnosis of RDD is challenging due to its rarity and wide variety of clinical manifestations.

RDD generally follows a benign course, but recurrences and poor outcomes have also been reported [4]. Currently, there are no established guidelines for managing Rosai–Dorfman disease (RDD). Various treatment options, including corticosteroids, surgery, radiotherapy, chemotherapy, or targeted therapy, have shown variable results. There are limited data available on radiation therapy regarding doses, fractionation, technique, and effectiveness in RDD [5].

In this context, we present a rare case of nasal RDD in a 54-year-old female refractory to systemic corticosteroid therapy and transnasal endoscopic resection that showed complete remission to localized radiotherapy.

## 2. Case Presentation

A 54-year-old female patient with no prior medical history presented to our ENT Department with progressive nasal obstruction, loss of smell, and occasional epistaxis for almost 1 year. Notably, she did not report any appetite changes, weight loss, shortness of breath, night sweats, changes in bowel habits, voice, or vision. Her past medical and family histories were non-significant, and she reported no smoking or alcohol consumption. A biopsy in another medical unit revealed a nonspecific mixed inflammatory reaction.

**Figure 1:**
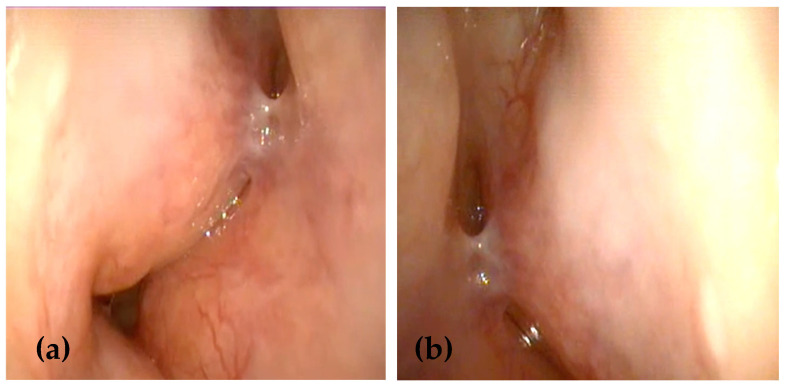
(**a**,**b**) Nasal endoscopy examination showing multiple submucosal swellings with a friable surface originating from the nasal septum and inferior turbinates in both nasal cavities, being more extensive on the left side.

Nasal endoscopy revealed extensive submucosal swellings, yellowish polyp-like space-occupying lesions with a friable surface, originating from the nasal septum and inferior turbinates in both nasal cavities, more extensive on the left side (Figure 1). The remainder of the ENT examination was unremarkable. No enlarged lymph nodes were detected based on physical examination or ultrasonography, and there was no evidence of systemic illness. A battery of tests was performed for further evaluation, including a complete blood count, ESR, serum electrolytes, renal function tests, chest X-ray, and imaging tests.

**Figure 2:**
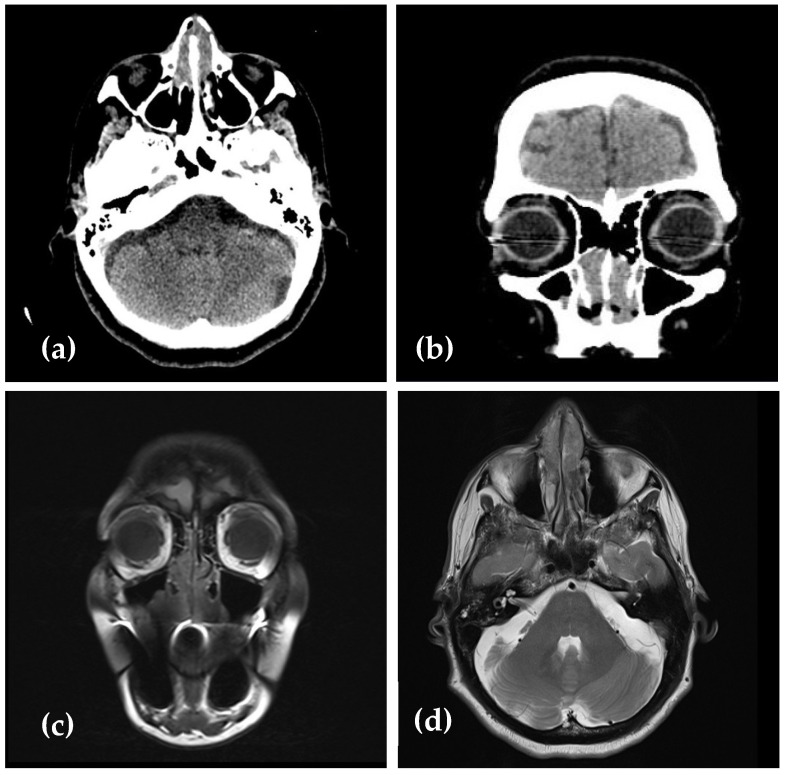
(**a**,**b**) Computed tomography scans of the head in the coronal and axial planes showing multiple, homogeneous, hypo-intense polypoid masses, which narrowed the nasal passages more extensively on the left side, obstructing the left choanal arch, without sinus involvement or bone erosion. (**c**,**d**) Coronal and axial T1-weighted images showing iso-intense to slightly hypo-intense lesions compared to gray matter.

Computed tomography (CT) of the sinuses revealed multiple, homogeneous, hypo-intense polypoid masses, which narrowed the nasal passages more extensively on the left side, obstructing the left choanal arch, without sinus involvement or bone erosion (Figure 2a,b).

Subsequent magnetic resonance (MR) of the head showed the lesions to be predominantly iso-intense to slightly hypo-intense on T1- and T2-weighted images compared to gray matter (Figure 2c,d).

**Figure 3:**
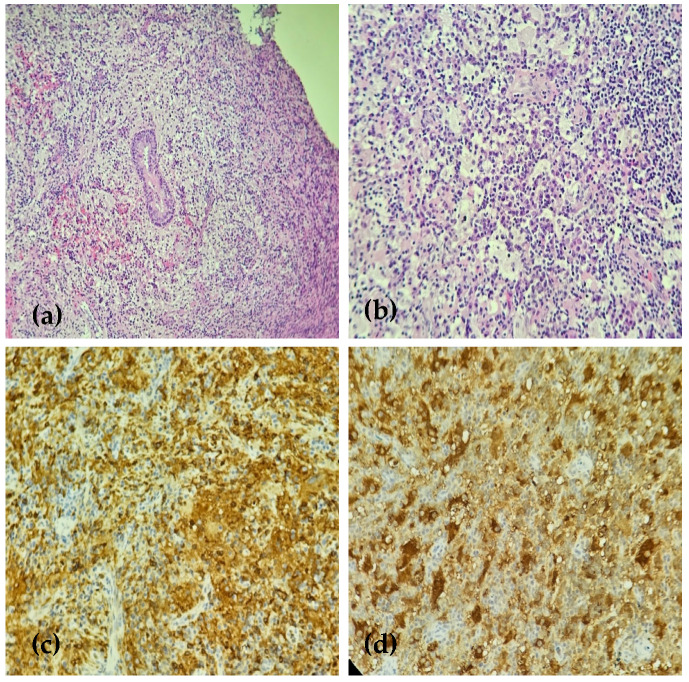
(**a**) HE 10×: Nasal-type mucosa showing massive inflammatory infiltrate consisting of numerous large pale histiocytes, small lymphocytes, and plasma cells. (**b**) HE20×: large histiocytes engulf intact small lymphocytes and plasma cells, a phenomenon called emperipolesis, which is fairly specific to RDD. (**c**) CD163 20×: The large histiocytes are positive for CD163. (**d**) S100 20×: The large histiocytes strongly co-express the S100 protein, which is characteristic of RDD.

Due to the nonspecific previous pathological examination, a new biopsy was performed, and most of the diseased tissue was excised for a larger tissue sample to achieve an accurate diagnosis. Pathologic examination showed polypoid edematous connective tissue covered by respiratory epithelium with subepithelial infiltrates of lymphocytes, plasma cells, and focally, some foam cells, a few eosinophils, and mast cells. Large histiocytes with aggregates of lymphocytes and nuclear debris in the cytoplasm were seen (Figure 3a,b). The immunohistochemical examination showed histiocyte positivity for S100 protein, CD163, and CD68, and negativity for CD1a (Figure 3c,d). These findings confirmed a diagnosis of sinus histiocytosis with features consistent with Rosai–Dorfman disease.

A complete computed tomography (CT) survey, including neck, thorax, abdomen, and pelvis, was routinely performed as part of the diagnostic evaluation of RDD, which showed no lymphadenopathy or other extranodal sites of involvement. A final diagnosis of nasal primary RDD without cervical lymphadenopathy was made. After pathologic diagnosis, the patient received systemic steroid therapy (oral prednisone 40 mg daily). The patient initially achieved a complete resolution of the lesions after 3 months of treatment, and the steroids were tapered down slowly. However, after a 4-month symptom-free period, the woman returned with worsening nasal obstruction. Nasal endoscopy revealed the same polyp-like submucosal swellings in both nasal cavities, narrowing the nasal passages. At this point, a multidisciplinary meeting with a radiotherapist and an oncologist was held regarding the treatment plan. It was decided to proceed with a secondary endoscopic debulking surgery followed by localized radiotherapy. The goal was to provide long-term symptom control while maintaining low levels of organ toxicity, considering that RDD is a benign disorder.

The surgery started by debulking the tumor using a microdebrider to increase space for instrumentation. We performed a complete resection of the inferior turbinate using Heymann forceps and the microdebrider. A Cottle dissector was used to separate the bone from the lateral wall of the nasal cavity. The lesions originating from the nasal septum were carefully resected without compromising the osteocartilaginous structure. Monopolar electrocautery was used to control bleeding and secure hemostasis. Intraoperative and postoperative periods were uneventful.

**Figure 4:**
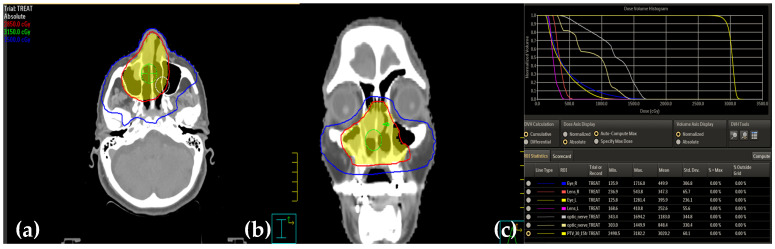
Three-dimensional conformal radiotherapy plans in the coronal (**a**), axial (**b**), and sagittal (**c**) views. Gross tumor volume boost delivered to the blue-shaded area in the isodose curves.

The choice of dose and fractionation was made based on several factors, including the patient’s age, overall condition, and social aspects. She received fractionated low-dose radiotherapy with a total dose of 30 cGy administered in 2 cGy daily fractions (30 cGy/15 fractions) (Figure 4). The treatment was well tolerated. No analgesics were required. According to the Radiation Therapy Oncology Group (RTOG) rating system, the patient’s symptoms were classed as grade 1 acute radiation-related effects on the nose. No skin toxicity was observed during the treatment. One week after the treatment, the nasal endoscopy revealed slight redness of the nasal mucosa and thicker secretions, with no recurrent disease.

**Figure 5:**
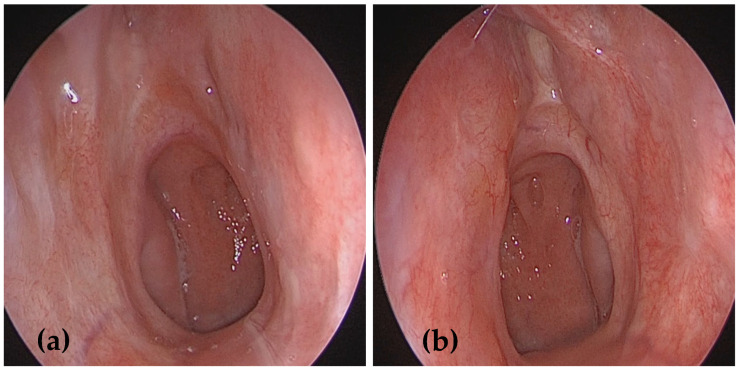
(**a**,**b**) A 36-month follow-up nasal endoscopy showed no evidence of residual tumor or local recurrence with normal healing of the nasal mucosa.

Thus, in our case, the radiation therapy successfully achieved long-term remission. The patient was followed up in the outpatient clinic. At the 3-year postradiotherapy visit, no evidence of tumor or local recurrence was observed during nasal endoscopy, with normal healing of the nasal mucosa (Figure 5). The postoperative scan was not deemed necessary in this case, as the disease was limited to the nasal cavity, without extension to the paranasal sinuses, a fact clearly seen in the initial imaging.

The possibility of relapse was thoroughly explained to the patient, who continues to attend regular follow-up visits for ongoing monitoring.

## 3. Discussion

RDD is a rare disorder with a prevalence rate of 1 per 200,000 population and approximately 100 new cases diagnosed annually in the United States [6]. It is found typically among children and young people, with an average age of 20.6 years and a male-to-female ratio of 1.4:1 [5].

The etiology of RDD is still poorly understood. Viral infections including herpes virus 6, Epstein–Barr virus, HIV, parvovirus B19, and cytomegalovirus have been associated with RDD. Still, none of these have been confirmed [7].

Recent studies discovered specific mutations in the MAPK/ERK signaling pathway, including BRAF, MAP2K1, KRAS, and NRAS. These findings indicate that genome instability may be one factor leading to RDD [8,9]. Garces et al. [10] found that in RDD, mutations were frequently associated with head and neck lesions. Germline mutations in SLC29A3 have been identified in patients with familial RDD [11]. The spectrum of SLC29A3-related diseases also known as histiocytosis-lymphadenopathy plus syndrome includes Faisalabad histiocytosis, Rosai–Dorfman disease, H syndrome, and pigmented hypertrichotic dermatosis with insulin-dependent diabetes (PHID) [11]. Additionally, heterozygous germline mutations in the FAS gene associated with autoimmune lymphoproliferative syndrome (ALPS) type I may be linked to RDD [12]. Some extranodal forms of RDD, such as those involving the colon, lungs, or liver, are associated with IgG4-RD [13].

In approximately 10% of cases, RDD coexists with immunological disorders, such as idiopathic juvenile arthritis, lupus, or autoimmune hemolytic anemia [14]. Moreover, histological characteristics of RDD have been observed in patients with both types of lymphoma, when RDD may occur before or after lymphoma, or coexist in the same lymph node [15].

Rosai–Dorfman disease typically presents with massive, painless swelling of the lymph nodes, particularly in the neck, often accompanied by systemic symptoms such as weight loss, night sweats, or fever. RDD can also manifest as extranodal disease, seen in approximately 43% of all cases, and is more frequent in older patients. Common extranodal sites include the skin (20.8%), lower respiratory tract (22.3%), eyelid/orbit (22.3%), and larynx (16.7%) [16]. RDD can also affect other head and neck sites, such as the salivary glands, pharynx, larynx, thymus, and thyroid gland, producing mass effect-related symptoms [17]. Generally, lymphadenopathy is associated with extranodal involvements, but solitary extranodal presentations have also been reported. However, the occurrence of RDD affecting the nasal cavity is relatively rare (11%) [18,19].

Patients with nasal involvement in Rosai–Dorfman disease (RDD) often present with nonspecific symptoms such as nasal obstruction, epistaxis, deformity of the nasal dorsum, facial asymmetry, sensation of fulness in the ears, rhinitis, and sinusitis [4]. The most common clinical findings on nasal endoscopy are extensive reddish-yellow-brown polyp-like lesions. Patients may present with fever, leukocytosis, neutrophilia, elevated erythrocyte sedimentation rate (ESR), anemia, and polyclonal hypergammaglobulinemia [20].

Imaging and nasal endoscopy may mimic nasal tumors, leading to potential misdiagnosis. Radiological imaging is essential in diagnosing and evaluating the extent and location of RDD lesions. RDD can present as single or multiple masses in different anatomical locations, making imaging necessary for accurate diagnosis and treatment planning. In children, initial investigations typically include a chest X-ray with neck and abdominal ultrasounds. For older patients, CT scans of the neck, chest, abdomen, and pelvis are recommended to evaluate the extent of the disease. FDG-PET/CT scans can also be used. To reduce radiation exposure in children, whole-body MRI is preferred over CT scans. Extranodal RDD, originating from the nasal cavity, presents nonspecific imaging findings, such as mucosal thickening or a soft tissue mass filling the nasal cavity or sinuses. However, osteosclerosis and bone erosion have been documented in some cases. Notably, the enhancement pattern of the RDD mass can be similar to tumor lesions, leading to misdiagnosis. In our case, the initial nasal endoscopy and preoperative imaging raised the suspicion of lymphoma [21].

Blood tests should include a metabolic panel, full blood count, sed rate (ESR), C-reactive protein test (CRP), and quantitative immunoglobulin levels. Serologies for HIV and hepatitis B and C are recommended to exclude associated diseases. Screening for autoimmune diseases such as lupus or juvenile arthritis is recommended as well.

Definitive diagnosis of RDD relies on histopathological and immunohistochemical examinations of tumor tissue. The hallmark histopathological feature of RDD is emperipolesis, characterized by large histiocytes with abundant pale cytoplasm that contain intact lymphocytes [22]. However, emperipolesis is not always present, particularly in extranodal lesions, and can also be seen in other histiocytic disorders, such as juvenile giant cell granuloma, lipoid granulomatosis (Erdheim–Chester disease), and malignant histiocytoses. Extranodal lesions tend to present more fibrosis, fewer RDD histiocytes, and less emperipolesis compared to nodal involvement [23].

Despite the characteristic histological features, RDD diagnosis can be challenging and requires clinical correlations. The primary differential diagnoses based on fine-needle aspiration cytology (FNAC) or external lymph node biopsy include other histiocytic disorders such as chronic inflammation, Erdheim–Chester disease, Langerhans cell histiocytosis, reactive lymphoid hyperplasia, and tuberculosis. Reactive sinus histiocytosis shows loose aggregates of histiocytes and infiltrates of germinal center cells, reactive lymphocytes, immunoblasts, and tangible body macrophages without emperipolesis. This can be misdiagnosed at times as reactive lymphadenopathy. Cytologically, Langerhans cells have grooved nuclei, and the background may contain eosinophilic microabscesses, which differentiate them cytologically from RDD, in addition to positivity for CD1a. In contrast, “tuberculous lymphadenitis” is characterized by epithelioid cell granulomas and caseous necrosis, which are not seen in RDD [24]. Other histological mimics of RDD include those of Gaucher’s disease, metastatic carcinoma, melanoma, histiocytic sarcoma, and lymphoma (both Hodgkin and non-Hodgkin lymphoma) [25].

The immunohistochemical examination is characterized by positivity for S100 protein and CD68, and inconstant expression of CD163 and CD14. Importantly, RDD histiocytes show negative staining for CD1a and CD207, which distinguishes RDD from Langerhans cell histiocytosis (LCH) [26].

Diagnosis of RDD can be particularly difficult in extranodal cases due to its variable pathological features and heterogeneous clinical manifestations. In our case, the first histopathological examination performed in another medical unit revealed chronic inflammation. Thus, biopsy specimens that show nonspecific inflammation, including lymphoid aggregates, plasma cells, and histiocytes, should raise concern for RDD. Integrating clinical, radiological, and histopathological findings is essential for achieving an accurate diagnosis, especially in challenging cases with extranodal presentations.

Managing RDD is challenging. According to the 2018 consensus, there is no established guideline for Rosai–Dorfman disease (RDD), and management should be personalized depending on the severity of the disease and the patient’s response to previous treatments [5].

Because of the benign nature of the disease and its self-limiting course, many patients without complicated lymphadenopathy or organ dysfunction do not require treatment and can allow the disease to run its natural course [5].

Therapeutic options, including corticosteroids, surgery, radiotherapy, chemotherapy, and targeted therapies, have shown variable results. Surgical interventions are usually limited to biopsy for histological confirmation, followed by observation in the majority of the cases. Surgical resection is often recommended for lesions that significantly affect quality of life or vital organ function [20]. In cases of unifocal disease, complete resection may offer a curative outcome. Debulking procedures are sometimes necessary to address complications such as airway obstruction, spinal cord compression, or large lesions that compromise organ function. For sinonasal RDD, endoscopic sinus surgery (ESS) has been effective in improving symptoms, with minimal complications and prolonged remission in some cases. In a case series, Ku et al. [27] reported two nasal RDD patients with no recurrences following endoscopic resection alone. However, complete surgical removal is often challenging. CO_2_ laser therapy has been suggested to reduce the risk of recurrence in nasal RDD. For patients with extensive, recurrent, or progressive RDD affecting vital organ functions, surgical debulking may be necessary. In severe cases involving the upper airway, tracheostomy may be required to maintain an open airway and relieve symptoms [25]. While surgical resection is often effective in alleviating symptoms, local recurrence or disease progression may occur after surgery. The specific factors influencing recurrence and the frequency of sinonasal RDD remain unclear. In multifocal RDD, surgical resection should be limited to bulky lesions that cause neurological symptoms or significant organ dysfunction [5].

Adjuvant therapies, including radiotherapy, chemotherapy, and steroids, have produced mixed results. For patients with progressive symptoms, different drug therapies have been attempted. Antitubercular drugs and antibiotics have not been effective [28]. On the other hand, corticosteroids are often used, leading to symptom improvement and, occasionally, complete remission. The optimal dose and duration of steroid treatment remain unclear, but tapering after achieving a response appears to be a reasonable approach. The effective corticosteroid doses reported in the literature range from 40 to 70 mg of prednisolone. Dexamethasone has also been effective in some cases, with 8 to 20 mg daily doses. One viable treatment strategy is to use steroids until the best response is observed, followed by a slow taper [5]. It is essential to monitor for potential steroid side effects, although they are generally well tolerated. Steroids have failed in some instances, including those involving the orbit, trachea, kidneys, or soft tissues. Relapses of RDD lesions have also been reported shortly after treatment is stopped. In a series of patients described by Komp et al. [29], partial or complete remission was observed in 4 of 36 patients, while 13 others experienced symptom improvement. In our case, considering the patient’s age and comorbidities, a 40 mg dose of prednisolone was prescribed. The lesion responded well to the medication, but after tapering off the medication, the lesions reappeared. From our clinical experience and based on the literature, extranodal RDD generally does not show a lasting response to steroid therapy alone, highlighting the need for additional or alternative treatments in such cases.

Chemotherapy (single agents or combinations) has shown limited success and may be considered for patients with disseminated or multifocal RDD, or when surgery and radiotherapy are ineffective. The efficacy of different chemotherapeutic agents varies widely [29,30]. These agents inhibit the function of monocytes by suppressing the synthesis of interleukin-6 (IL-6), interleukin-1 beta (IL-1 β), and tumor necrosis factor alpha (TNF-α) and should be considered in severe or refractory cases where the potential benefit outweighs the myelosuppressive toxicity. However, aggressive systemic therapy is not typically recommended for localized RDD, as it may not provide significant benefits [25].

Radiotherapy shows modest efficacy in treating RDD. It is often used in cases of refractory soft tissue or orbital bone disease that compromise vision. Radiotherapy is also used for resistant airway obstruction or to alleviate local symptoms [5]. In most studies, however, the dose of radiation applied was not specified, so the data can only be interpreted with limitations. Ten patients achieved partial or complete remission after radiotherapy [29,31]. Some studies suggested that low-dose radiation combined with surgery or corticosteroids provides better local control. However, radiation is mainly recommended for palliative care in patients with multifocal, symptomatic disease. There are no established standard doses for radiotherapy in RDD. However, doses ranging from 30 to 50 Gy have been used in clinical practice [19]. Further research is needed to define optimal dosing protocols and improve outcomes for patients who are undergoing radiotherapy for RDD.

In our case, the patient achieved complete remission of the lesions, and at the 3-year follow-up postradiotherapy visit, no evidence of tumor or local recurrence was observed. The patient continues to attend regular follow-up appointments.

Immunomodulatory therapies such as TNF-α inhibitors (Thalidomide, Rituximab) have shown promising results in managing RDD [5,32]. Moreover, tumor sequencing and targeted therapies, such as MEK inhibitors, have gained popularity in the last few years and may have clinical applications in RDD diagnosis and management, though the evidence remains limited [5,33]. Exploring these therapies in refractory RDD cases may improve outcomes for patients with a poor prognosis.

In our opinion, based on the limited experience with the case discussed, and also based on reviewing the literature, conservative treatment should be considered first, using a high dose of prednisolone. If steroid therapy fails within a reasonable timeframe and symptoms persist, endoscopic endonasal tumor debulking should be considered next. Adjuvant therapy should be considered to reduce the risk of recurrence. For patients who do not respond to steroids or cannot tolerate high doses, low-dose radiotherapy might be an alternative, although its effectiveness is uncertain, according to the literature.

Prognosis is generally favorable, especially for nodal and cutaneous forms, which are often self-limiting [34]. However, some patients have an unpredictable disease course, with alternating periods of remission and reactivation that may last years.

The overall survival of patients with RDD is not well defined, but large lesions involving multiple organs have been associated with poor postoperative outcomes [35]. The prognosis for RDD varies depending on the presentation. Research has shown a 14.3% mortality rate among patients with extranodal RDD in the head and neck region and a 4.8% mortality rate among those with nasal RDD [36,37]. In the largest series reported by Foucar et al. [28], 7% of patients (17 out of 238) died from complications of the disease. A later review by Pulsoni et al. [38] showed a 12% mortality rate (10 out of 80 patients), with those having multifocal or extranodal involvement (mainly affecting the kidneys, liver, or lungs) showing poorer outcomes. For these patients, intensive treatments such as chemotherapy or targeted therapies may be necessary. Exploring these therapies in refractory RDD cases may improve outcomes for patients with a poor prognosis. This highlights the need for individualized treatment plans and ongoing research to refine therapeutic strategies for challenging RDD cases.

Considering the rarity of RDD in the sinonasal tract, this case offers valuable insights into the literature regarding differentiating RDD from other lymphoproliferative disorders in terms of histopathological and immunohistochemical findings. It highlights the challenges posed by the lack of standardized treatment guidelines and underscores the importance of multidisciplinary care in managing rare lymphoproliferative disorders. Additionally, this case emphasizes the need for further exploration of novel therapies, such as immunotherapy and targeted treatments, which may improve outcomes in similar RDD cases. This case contributes to the limited literature and may guide future treatment protocols. Reporting rare cases such as this one contributes to improving the knowledge base of RDD.

## 4. Conclusions

Rosai–Dorfman disease (RDD) is a rare and heterogeneous disorder that poses significant diagnostic and therapeutic challenges. The evaluation and management of RDD often require a multidisciplinary approach.

RDD is a rare proliferative disorder that is often self-limiting. Its evaluation requires a thorough medical history and clinical examination, imaging studies, and blood tests to assess the extent of the disease. Many cases do not require treatment, but for symptomatic patients, treatment options include systemic corticosteroids, surgery, radiotherapy, and chemotherapy. In our case, complete remission was achieved after radiation therapy, highlighting the potential role of radiotherapy as an effective therapeutic option for relapsing or steroid-refractory cases.

For nasal involvement, endoscopic sinus surgery followed by radiotherapy may be an effective therapeutic option for relapsing or steroid-refractory cases.

Reporting of future cases treated with radiotherapy should include information about the technique, radiation dose, fractionation, toxicity, and outcome to contribute to the development of protocols for managing RDD.

## Data Availability

The data presented in this study are available on request from the corresponding author.

## List of Abbreviations

RDD	Rosai–Dorfman disease
SLC29A3	Solute carrier family 29 member 3
MAPK	Mitogen-activated protein kinase
ERK	Extracellular signal-regulated kinase
BRAF	V-Raf murine sarcoma viral oncogene homolog B
KRAS	Kirsten rat sarcoma 2 viral oncogene homolog
FDG-PET scan	Fluorodeoxyglucose-positron emission tomography
NRAS	Neuroblastoma ras viral oncogene homolog
MAP2K1	Mitogen-activated protein kinase kinase 1
IgG4-RD	Immunoglobulin G4-related disease
